# WHAT COMES AFTER BEING NAMED EMERGENCY CONTACT: EXPERIENCES OF CURRENT AND POTENTIAL SURROGATE DECISION-MAKERS

**DOI:** 10.1093/geroni/igae098.3392

**Published:** 2024-12-31

**Authors:** Maresi Berry-Stoelzle, Sarah Murray, Daniel Karr, Tammy Walkner, Bruce Alexander

**Affiliations:** Iowa City VA Medical Center, Iowa City, Iowa, United States; Iowa City VA Medical Center, Iowa City, Iowa, United States; Department of Veterans Affairs, Iowa City, Iowa, United States; Iowa City VA Health Care System, Iowa City, Iowa, United States; Iowa City VA Medical Center, Iowa City, Iowa, United States

## Abstract

Veterans have high rates of chronic health conditions than non-veterans. As their comorbidity progresses, recommended therapies are often more complex and may not contribute to the Veteran’s quality of life. During hospital admissions, a combination of medication and illness can result in the Veteran being unable to make decisions for themselves. Their designated Durable Power of Attorney (DPOA) or, if none is designated, the Emergency Contact/Next of Kin (EC/NoK) then makes these decisions. This mixed-methods study identified Veterans and their EC/NoK who had been hospitalized at a VAMC during the last year. Semi-structured interviews with EC/NoKs focused on identifying support needs, challenges of the health care system, perceptions of “goals of care” conversations, and the responsibilities of being a medical decision-maker. Questions were determined from prior literature on 1) experience of caregivers and 2) medical decision making for others. Four themes were identified via Consolidated Framework for Implementation Research: 1) when EC/NoK have higher social connections with the Veteran’s healthcare team, they report higher confidence and preparedness for their role; 2) most preparation consisted of conversations with the Veteran about terminal decisions, 3) EC/NoK with the highest self-report of preparedness reported previous experience with medical decision-making; 4) those whose self-reported lower confidence also reported higher levels of isolation and a lack of support in their caregiver role. These interviews with EC/NoK demonstrate opportunities to increase knowledge of medical decision-making and the opportunities to support EC/NoK’s in their role as DPOA’s within a healthcare system.

